# Splenic Injury in Sleeve Gastrectomy: Mechanisms, Risk Factors, and Management Strategies

**DOI:** 10.34172/mejdd.3341

**Published:** 2026-04-30

**Authors:** AmirHossein Latif, Amir Monshizadeh, Khosrow Najjari, Hossein Zabihi Mahmoudabadi, Ahmad Reza Soroush, Hamid Reza Soltani

**Affiliations:** ^1^Department of General Surgery, Dr. Shariati Hospital, Tehran University of Medical Sciences, Tehran, Iran; ^2^Department of Minimally Invasive Surgery, Tehran University of Medical Sciences, Tehran, Iran; ^3^Department of General Surgery, Sina Hospital, Tehran University of Medical Sciences, Tehran, Iran

**Keywords:** Bariatric surgery, Laparoscopic sleeve gastrectomy, Splenic injury, Clinical management

## Abstract

Splenic injury represents a rare but significant complication during laparoscopic sleeve gastrectomy that can lead to considerable morbidity if not promptly diagnosed and managed. This comprehensive review aims to elucidate the mechanisms underlying splenic injuries, assess patient- and procedure-specific risk factors, and evaluate the clinical presentation and management strategies for these events. A literature search was performed across major databases to identify relevant articles published over the past decades. Evidence suggests that factors such as technical nuances during dissection, the handling of short gastric vessels, and patient-specific anatomical variations contribute to the risk of splenic injury. Management ranges from conservative monitoring to surgical interventions, such as splenectomy or embolization, depending on the severity of the injury. Furthermore, this review discusses preventive measures and techniques to minimize these risks, offering important insights for bariatric surgeons to enhance patient outcomes.

## Introduction

 Obesity has reached epidemic proportions globally, affecting millions of individuals and contributing to a myriad of comorbidities such as type 2 diabetes, cardiovascular disease, and metabolic syndrome.^1,2^ Bariatric surgery has emerged as one of the most effective interventions not only for achieving significant weight reduction but also for markedly improving obesity-related conditions.^3,4^ Among the various bariatric procedures, laparoscopic sleeve gastrectomy (LSG) has gained substantial popularity because of its technical simplicity, favorable risk profile, and demonstrable long-term benefits.^5^

 Laparoscopic sleeve gastrectomy involves resecting a large portion of the stomach along its greater curvature to create a tubular gastric remnant. Although this procedure has significantly advanced bariatric care, it is not without complications. One particularly challenging, yet often under-recognized, complication is splenic injury.^6^ Due to the anatomical proximity of the spleen and the short gastric vessels, surgical maneuvers during the mobilization of the greater curvature (which may include inadvertent traction or inadvertent injury during vessel ligation) can lead to contusions, lacerations, or vascular compromise of the spleen.^7^ Even though the reported incidence of splenic injury in LSG cases is relatively low, ranging from 0.2% to 1%, the potential for significant morbidity underscores the need for a comprehensive understanding of its underlying mechanisms and appropriate management strategies.^6-8^

 This narrative review aims to synthesize evidence from recent studies to provide a detailed assessment of splenic injury in the context of LSG. The objectives of this article are to (i) elucidate the surgical and patient-specific factors contributing to splenic injury, (ii) analyze the clinical presentation and diagnostic challenges associated with these injuries, (iii) evaluate both conservative and surgical management options, and (iv) propose evidence-based preventive measures to inform and enhance bariatric surgical practice.^9,10^ By consolidating the existing literature, this review seeks to increase clinician awareness, support timely intervention, and guide future research in this critical area.^11^

## Mechanisms of Splenic Injury

 The occurrence of splenic injury during LSG is multifactorial and reflects a complex interplay between anatomical vulnerabilities, surgical technique, and individualized patient characteristics (Figure 1).

**Figure 1:**
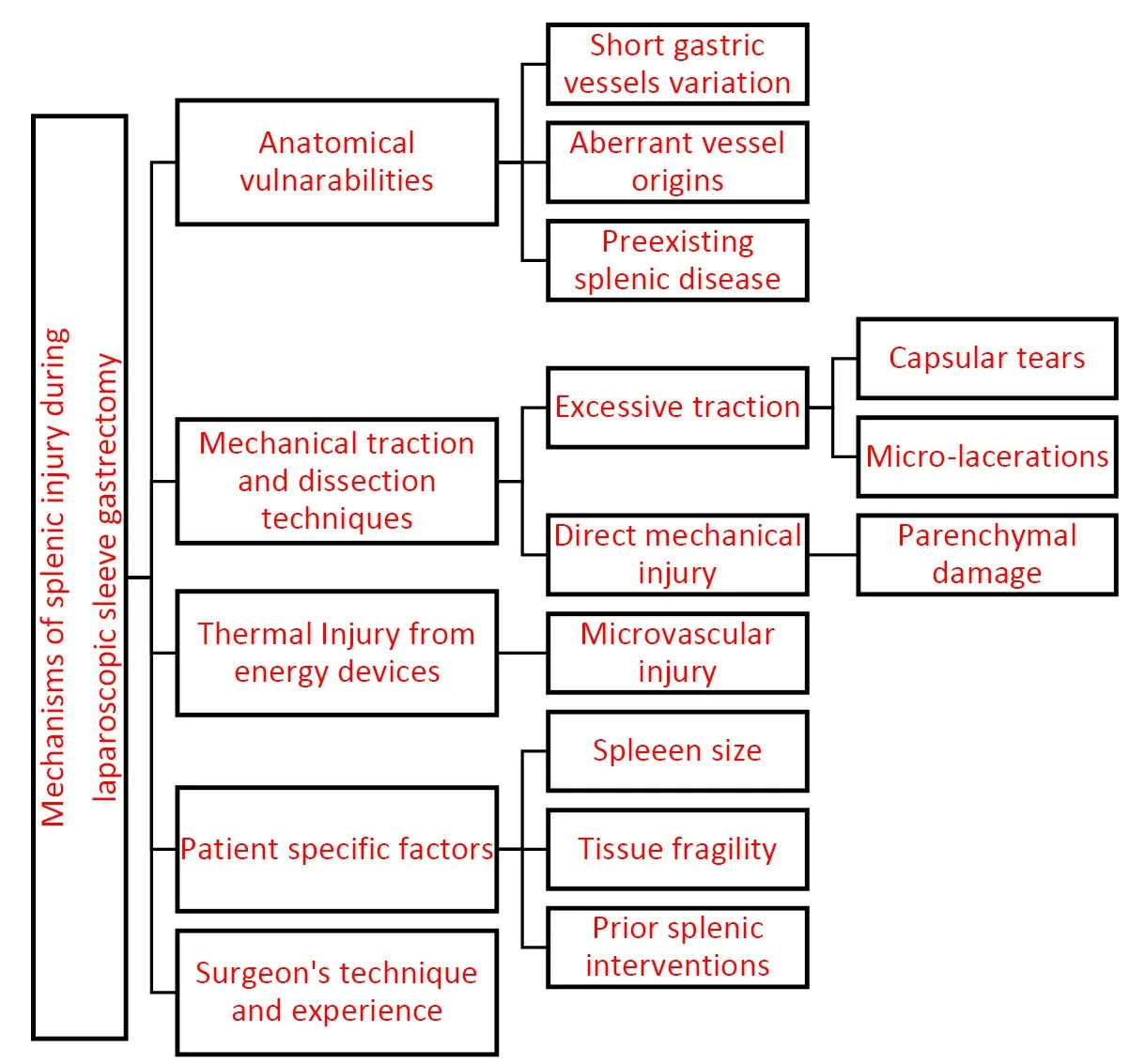
Mechanisms of splenic injury during laparoscopic sleeve gastrectomy

## Anatomical Vulnerabilities

 The spleen is a highly vascular organ with a delicate capsule and is closely associated with the stomach via the gastrosplenic ligament, which houses the short gastric vessels.^12^ During LSG, dissection of the greater curvature necessitates division of these vessels to mobilize the stomach; however, patient-to-patient variation in their length, branching, and caliber can predispose the spleen to inadvertent trauma.^12,13^ Some patients exhibit a predominance of specific short gastric branches that supply a larger portion of the splenic parenchyma, thereby amplifying the risk of ischemia when these vessels are ligated.^13^ Preoperative imaging studies, such as computed tomography (CT) angiography, have highlighted these anatomical differences, suggesting that individual vascular mapping might be beneficial for risk assessment.^12^

## Mechanical Traction and Dissection Techniques

 Intraoperative maneuvers significantly contribute to the risk profile. Adequate exposure during the resection of the greater curvature often requires traction of the stomach, which can inadvertently transmit force to the splenic hilum and capsule. Excessive or misdirected traction has been reported to cause micro-lacerations or capsular tears, which may not be immediately apparent but can lead to delayed hemorrhage or splenic infarction.^14^ Moreover, aggressive or imprecise dissection near the gastrosplenic ligament increases the likelihood of direct mechanical injury. Case series have indicated that even seemingly minor parenchymal damage during mobilization can evolve into significant complications, necessitating urgent interventional measures.^14,15^

## Thermal Injury from Energy Devices

 Advanced energy devices such as bipolar electrocautery and ultrasonic shears are routinely used during LSG to control bleeding and facilitate dissection. Nonetheless, the thermal energy emitted by these instruments can extend beyond the target area, inadvertently causing collateral damage to adjacent splenic tissue. Thermal spread can result in microvascular injury, leading to parenchymal necrosis, localized infarction, and in rare instances, eventual splenic rupture.^16^ The risk is particularly pronounced when energy devices are used in close proximity to the splenic capsule or when excessive energy is applied during vessel sealing.

## Patient-Specific and Technical Factors

 Apart from inherent anatomical challenges, the surgeon’s technique and the patient’s unique physiological attributes play an essential role. For instance, variations in splenic size and tissue fragility—as seen in patients with underlying inflammation or prior splenic interventions—can heighten the risk of injury during routine mobilization.^15^ In particular, patients with a history of splenic artery embolization may have compromised collateral circulation; in these cases, even meticulous ligation of the short gastric vessels could precipitate splenic infarction.^2^ Hence, intraoperative assessment of splenic perfusion—using tools such as indocyanine green (ICG) fluorescence imaging has been advocated as a useful measure to guide the surgical approach and minimize injury.^2^ Additionally, the surgeon’s experience and adherence to refined dissection protocols are vital in mitigating the inherent risks.

## Risk Factors

 It should be considered that several factors may have role in the occurrence of splenic injury during LSG. Multiple studies have identified a combination of patient-specific and procedure-related factors that can increase the risk of such complications.

## Patient-Specific Factors

###  Obesity and High Body Mass Index

 Morbid obesity, by definition, is characterized by an elevated body mass index (BMI) and is often accompanied by excessive intra-abdominal adipose tissue. This increased adiposity can obscure normal anatomical landmarks and require more forceful retraction to obtain adequate exposure. The need for vigorous manipulation places additional stress on the splenic capsule and its vascular attachments, making the spleen more vulnerable to injury during dissection.^17,18^

## Previous Abdominal Surgery and Adhesions

 Previous abdominal procedures are predisposing factors to the development of adhesions. These fibrous bands can distort normal anatomical planes, complicating the mobilization of the stomach and increasing the risk of inadvertent splenic trauma. Adhesions may tether the spleen or surrounding structures, thereby reducing surgical flexibility and increasing the risk of direct laceration or indirect injury from increased tension.^14,15^

## Advanced Age and Comorbidities

 Although age itself has not been consistently reported as an independent predictor of splenic injury, advanced age can be associated with reduced tissue resilience and elasticity. Additionally, comorbid conditions such as hypertension, diabetes, or other vascular pathologies may compromise tissue integrity and impair healing, thereby enlarging the margin of error during surgical manipulation.^18^ Variability in splenic tissue quality and concurrent systemic conditions may predispose these patients to intraoperative injury even with standard dissection techniques.

## Procedure-Related and Technical Factors

###  Excessive Traction and Aggressive Dissection

 During LSG, the mobilization of the greater curvature requires careful retraction of the stomach. However, excessive or misdirected traction—particularly to the area of the gastrosplenic ligament—may cause micro or even macro capsular lacerations in the spleen.^14^ Numerous case series have documented that even minor parenchymal injuries, which may initially be unnoticed, can evolve into significant complications such as delayed hemorrhage or splenic infarction.^14,15^

## Thermal Injury from Energy Devices

 Microvascular damage and subsequent parenchymal necrosis may occur due to inadvertent thermal injury when these energy devices are used in close proximity to the splenic capsule or adjacent short gastric vessels.^16^ This thermal insult, often compounded by aggressive energy application, can precipitate localized infarction or even delayed rupture of the spleen.

## Anatomical Variability and Vascular Architecture

 Another key consideration is the inherent variability in the anatomy of the gastrosplenic ligament and the short gastric vessels. Research has highlighted considerable inter-individual differences in vessel size, branching patterns, and overall vascular distribution.^13^ Not to precisely recognize a dominant vessel or variant anatomy may put the spleen at risk of injury.

## Surgical Approach and Intraoperative Technique

 Beyond the intrinsic technical challenges of the procedure, additional risk is associated with certain intraoperative decisions. Suboptimal port placement or inadequate visualization due to improper patient positioning can force the surgeon to work at awkward angles, increasing the likelihood of inadvertent splenic strain or injury.^15^ Furthermore, the learning curve associated with laparoscopic techniques is nontrivial; surgeons with less experience may inadvertently apply excessive force or misinterpret anatomical relationships, thereby increasing the risk of splenic complications.^14,15^ The adoption of adjunctive technologies—such as ICG fluorescence imaging—has been proposed not only to enhance visualization but also to assess splenic perfusion in real time, reducing the likelihood of injury.^19^

## Clinical Presentation

 Splenic injury during LSG can manifest in a broad spectrum of clinical presentations depending on the nature and severity of the insult. These presentations may be broadly grouped into acute, delayed/subacute, and rare spontaneous events, each requiring tailored diagnostic and management approaches.

## Acute Presentations

 Patients with significant intraoperative splenic injury typically present in the early postoperative period with acute left upper quadrant (LUQ) pain that can radiate to the left shoulder. Such pain is often accompanied by clinical indicators of intra-abdominal hemorrhage (e.g., hypotension, tachycardia, and a sudden drop in hemoglobin levels).^20^ CT is usually the imaging modality of choice in these cases, as it may reveal subcapsular hematomas, active contrast extravasation, or even an altered splenic contour in cases of severe injury.^21^

## Delayed and Subacute Presentations

 Not all splenic injuries become clinically evident immediately. Minor parenchymal or vascular insults can evolve into delayed complications:

## Splenic Abscess

 Some patients develop a splenic abscess days to weeks after surgery. These patients typically present with persistent fever, vague LUQ discomfort, and general malaise. CT imaging in these cases frequently shows hypodense lesions with irregular margins, which are suggestive of abscess formation or evolving infarction.^20,22^

## Splenic Infarction

 In cases of minor vascular compromise, a segmental splenic infarction may occur. Often, these infarcts begin as subclinical lesions that are identified incidentally on follow-up imaging examinations.^22^

## Spontaneous Rupture

 Spontaneous splenic rupture is a very rare and severe phenomenon. In such scenarios, patients who initially appear clinically stable may rapidly deteriorate, exhibiting signs of peritoneal irritation and overt shock secondary to rupture of the injured spleen.^21,23,24^ This unpredictable event underscores the importance of ongoing postoperative vigilance—even when early recovery seems uneventful.

## Management Strategies

 The management of splenic injury after LSG requires a tailored, multidisciplinary approach. The strategy must consider the patient’s hemodynamic status, the grade and extent of injury as identified on imaging, intraoperative findings, and the overall clinical scenario. Management options range from conservative observation and medical management to interventional radiology procedures and emergent surgical intervention. In addition, emerging techniques and rigorous postoperative follow-up protocols are essential components of an effective management plan (Figure 2).

**Figure 2:**
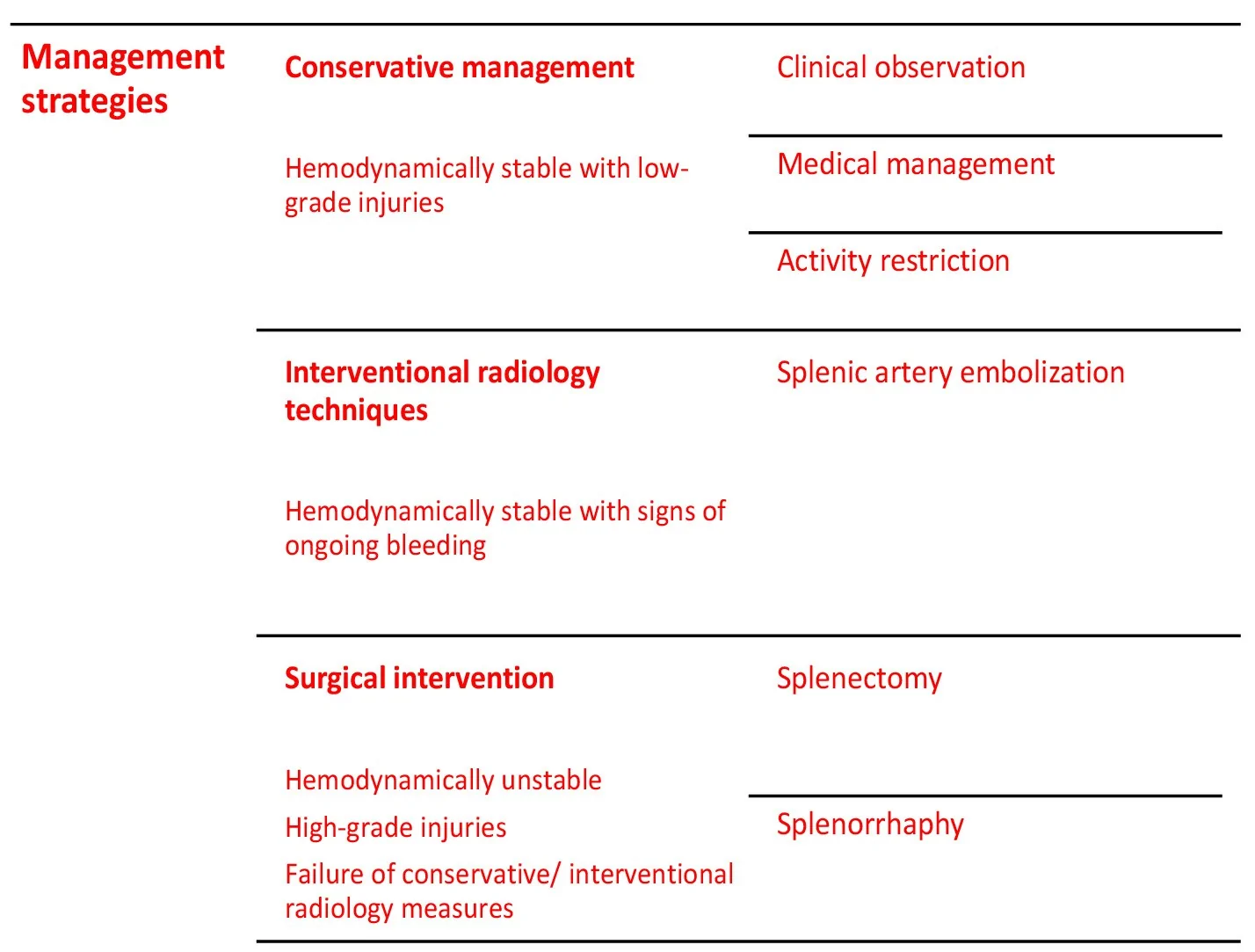
Management strategies in splenic injury during laparoscopic sleeve gastrectomy

## Conservative (Non-Operative) Management

 For patients who are hemodynamically stable with low-grade injuries (e.g., small subcapsular hematomas or contained lacerations with minimal parenchymal disruption), a conservative management approach is often considered:

## Observation and Supportive Care

 Clinical observation remains the cornerstone for managing low-grade splenic injuries. This involves admission to a monitored setting, serial examinations, and continuous monitoring of vital signs and hematologic parameters (e.g., serial hemoglobin/hematocrit checks). Repeat imaging (ultrasound or contrast-enhanced CT scan) is scheduled to assess the evolution of the hematoma or infarcted areas.^25-27^

## Medical Management

 Supportive measures include intravenous fluids, pain control, and, when indicated, prophylactic antibiotic therapy. In instances where minor vascular compromise is suspected, medical management helps prevent secondary infection and promote resolution of the injury.^25,27^

## Activity Restrictions

 Patients are generally advised to avoid strenuous activities to prevent exacerbation of the injury. Bed rest may be recommended during the acute phase until radiological evidence of stabilization is present.^25^

## Interventional Radiology Techniques

 Interventional radiology (IR) plays a critical role in the management of splenic injuries that involve active bleeding or localized vascular disruption, particularly in patients who remain hemodynamically stable yet show signs of ongoing hemorrhage:

## Splenic Artery Embolization

 Splenic artery embolization (SAE) has emerged as an effective, organ-preserving procedure that can control bleeding by selectively occluding injured vessels. This technique helps stabilize patients without resorting to immediate splenectomy, thus preserving immunologic function. Studies have demonstrated that partial embolization can reduce arterial inflow while maintaining some splenic perfusion through collateral networks (most notably, the short gastric and gastroepiploic vessels).^28,29^

## Technical Considerations and Imaging Guidance

 The procedure is typically performed under fluoroscopic or CT guidance. To determine the site and extent of vascular injury, an angiographic study is first undertaken. Embolic agents (such as coils, particles, or glue) are then deployed selectively. Post-procedure, patients require close observation for complications, including post-embolization syndrome (fever, pain, and transient leukocytosis), as well as potential abscess formation.^28,29^

## Advantages and Limitations

 While SAE is less invasive than surgery and offers a high rate of hemostatic success, it is not without risks. Complications may include splenic infarction beyond the targeted area, abscess formation, and rebleeding. Thus, patient selection is critical, and IR should be deployed in centers with both the expertise and capabilities for rapid follow-up.^28,29^ Despite the advantages of SAE, the threshold for its application is still unclear, and further studies are needed.

## Surgical Management

 Surgical intervention remains the definitive treatment for patients with high-grade splenic injuries or those who are hemodynamically unstable, or in whom nonoperative and IR measures have failed.

## Splenectomy

 In cases of massive hemorrhage or extensive parenchymal disruption, splenectomy (either laparoscopic or open) is commonly required. Laparoscopic splenectomy is favored when feasible due to its minimally invasive nature and related benefits (less postoperative pain, shorter hospital stay, and quicker recovery), but conversion to open procedure is often necessary when there is extensive bleeding or poor visualization.^24,30^

## Considerations for Splenectomy

 Surgeons must weigh the risks of loss of immunologic function—a factor that increases the long-term risk of overwhelming post-splenectomy infection (OPSI)—against the immediate need to control hemorrhage. It should be emphasized that the decision for surgical intervention should be made based on patient’s clinical status, available facilities, and accessible specialists. When splenectomy is performed, appropriate vaccinations (e.g., pneumococcal, meningococcal, and Haemophilus influenzae type b) are administered preoperatively (if possible) or in the early postoperative period.^24^

## Organ-Preserving Procedures (Splenorrhaphy)

 Although less common due to technical challenges and variable success rates, splenorrhaphy may be attempted in selected patients with moderate injury, especially when preservation of splenic function is critical (e.g., in pediatric populations or immunocompromised patients). This approach involves direct repair of the splenic capsule and parenchyma using techniques such as suturing or application of topical hemostatic agents.^24,30^

## Intraoperative Adjuncts

 Intraoperative imaging techniques, such as ICG fluorescence angiography, can help assess splenic blood flow and viability during repair attempts. These techniques aid in determining whether organ-preserving strategies are feasible versus if conversion to splenectomy is required.^31^

## Decision-Making in the Operating Room

 The choice between continued conservative management, IR, and surgical intervention depends on dynamic intraoperative findings and the patient’s evolving clinical status. A multidisciplinary team—including bariatric surgeons, trauma surgeons, interventional radiologists, and critical care specialists—ensures that rapid, evidence-based decisions can be made, optimizing outcomes.^24,30^

## Postoperative Management and Surveillance

 Vigorous postoperative care is essential regardless of the chosen management strategy:

## Intensive Monitoring

 Patients who have sustained splenic injury require admission to a high-dependency unit or intensive care setting for continuous monitoring of vital signs, laboratory parameters, and hemodynamic status. This is especially true for those managed nonoperatively or with IR interventions, given the risk for delayed hemorrhage or infection.^27^

## Serial Imaging

 Follow-up imaging studies (ultrasound or CT scans) are essential to monitor the evolution of hematomas, infarcts, or any developing complications such as abscess formation. Early identification of adverse changes may prompt timely intervention before clinical deterioration.^27^

## Vaccination and Infection Prophylaxis

 In patients undergoing splenectomy, post-splenectomy prophylaxis—including vaccinations—is critical. For patients managed with spleen-preserving techniques, clinical guidelines recommend similar follow-up to detect any functional deficits in splenic reserve.^32^

## Rehabilitation and Patient Education

 Patients are counseled on activity restrictions and the importance of adhering to follow-up schedules. They are educated regarding warning signs, such as new-onset fever, persistent abdominal pain, or changes in vital signs, which necessitate prompt medical evaluation.^27^

## Adjunct Strategies and Emerging Approaches

 Recent advances have led to several adjunct strategies that enhance management:

## Preoperative Planning

 Detailed preoperative imaging—such as CT angiography—helps map individual splenic vascular anatomy and identify patients at higher risk for injury. This allows for proactive surgical planning, including adjustments in technique to minimize splenic manipulation.^31^

## Intraoperative Imaging

 The use of real-time ICG fluorescence enables the surgeon to assess splenic perfusion after division of the short gastric vessels. This technique can help guide decisions regarding the need for splenic preservation versus immediate intervention.^31^

## Multidisciplinary Team Approach

 Integration of expertise from bariatric surgery, trauma surgery, interventional radiology, and critical care improves decision-making and patient outcomes. Regular multidisciplinary case reviews and adoption of evidence-based protocols facilitate the implementation of best practices.

## Research and Emerging Techniques

 Ongoing research into novel embolic agents, improved imaging modalities, and minimally invasive surgical techniques continues to expand the therapeutic armamentarium. Emerging therapies, such as targeted molecular hemostatic agents, hold promise for future management protocols.

## Preventive Measures

 Preventing splenic injury during LSG requires a proactive, multifaceted strategy that addresses the entire patient journey—from preoperative evaluation through intraoperative technique and rigorous postoperative monitoring. Comprehensive preventive measures are critical not only for reducing immediate morbidity but also for enhancing long-term outcomes.

## Preoperative Planning and Risk Stratification

 A cornerstone of prevention is meticulous preoperative risk assessment:

## Detailed Imaging and Anatomical Mapping

 Preoperative imaging with contrast-enhanced CT angiography or magnetic resonance angiography can delineate the anatomy of the gastrosplenic ligament and short gastric vessels. Identifying anatomical variations—such as aberrant vessel origins or unusually fragile splenic tissue—enables surgeons to tailor their approach.

## Risk Factor Identification and Patient Counseling

 Evaluation of patient-specific risk factors (e.g., high BMI, previous upper abdominal surgery, and comorbid conditions that may compromise tissue integrity) allows for stratification of risk. In high-risk patients, alternative bariatric procedures or modified dissection techniques may be considered. Preoperative discussions should include detailed counseling on potential complications and the need for prophylactic measures (including appropriate vaccinations if splenic compromise is anticipated).

## Intraoperative Techniques and Adjuncts

 Optimization of intraoperative technique is paramount to prevent iatrogenic splenic injury:

## Precise Dissection and Instrumentation

 To minimizes inadvertent traction on the spleen, dissection near the gastrosplenic ligament should be gentle and precise. Surgeons are advised to use meticulous electrosurgical techniques and advanced energy devices with controlled thermal spread. This careful approach helps avoid collateral thermal injury to the splenic capsule and underlying parenchyma.^14^

## Optimal Retraction and Exposure

 Utilizing proper retraction tools and techniques avoids excessive traction forces. Maintaining a proper angle and avoiding over-manipulation near the splenic hilum can reduce mechanical stress on splenic attachments.^14^

## Intraoperative Perfusion Assessment

 The use of ICG fluorescence imaging has emerged as a valuable adjunct. ICG facilitates real-time assessment of splenic perfusion after division of the short gastric arteries, ensuring that the organ retains adequate blood flow. This imaging modality provides immediate feedback, allowing the surgeon to modify the dissection if compromised perfusion is detected.^31^

## Adherence to Standardized Protocols

 Implementation of standardized surgical protocols—including checklists and surgical time-outs focused on critical anatomical landmarks—can ensure that every step is performed with due caution. Protocols may also include predefined criteria for limiting the use of energy devices in proximity to the spleen.

## Surgeon Training and Experience

 The surgeon’s expertise is a significant determinant of complication rates:

## Enhanced Training and Simulation

 Simulation-based training for bariatric procedures and cadaveric dissections can help surgeons refine their technique, especially with respect to identifying and managing anatomical variations. Training modules that focus on minimizing splenic traction and using advanced energy devices are highly recommended.

## Mentorship and Multidisciplinary Collaboration

 Senior surgeon mentorship, as well as collaboration with interventional radiologists and experienced bariatric teams, support knowledge transfer and adherence to best practices. Regular morbidity and mortality reviews and case conferences help disseminate lessons learned from splenic injuries.

## Postoperative Surveillance and Early Intervention

 Even with optimized intraoperative techniques, the risk of delayed splenic complications remains:

## Early Postoperative Imaging

 For high-risk cases or when intraoperative concerns arise, routine postoperative imaging (ultrasound or CT scans) can help identify evolving complications (e.g., splenic infarction or hematoma) before clinical deterioration occurs.^27^

## Close Clinical Monitoring

 Intensive monitoring of vital signs and laboratory parameters in the initial postoperative period facilitates early detection of complications. Patients should be educated about the warning signs of splenic compromise (persistent left upper quadrant pain, fever, or hemodynamic changes) to ensure prompt evaluation.^27^

## Future Directions and Emerging Approaches

 The incidence of splenic injury may be reduced in the future with improved strategies:

## Advances in Energy Devices

 Continued refinement of energy devices that deliver precise energy with minimal thermal spread is an active area of research. Adoption of such technology may further lower the risk of collateral damage to splenic tissue.

## Personalized Surgical Planning

 Integration of three-dimensional reconstructions of vascular anatomy from preoperative imaging may allow personalized surgical planning. This tailored approach could optimize dissection strategies and further reduce inadvertent injury.

## Conclusion

 Splenic injury during laparoscopic sleeve gastrectomy remains a rare but potentially life-threatening complication. This review has elucidated the multifactorial mechanisms underlying these injuries—from inherent anatomical vulnerabilities (such as aberrant or fragile short gastric vessels) and mechanical forces generated during aggressive dissection, to the thermal spread associated with certain energy devices.^12,16^ Moreover, the risk of splenic injury may be compounded by patient-specific factors, such as comorbidities, high BMI, and a history of abdominal surgery, as well as procedure-related factors, underscoring the need for individualized risk stratification.^15,17,18^

 Clinically, splenic injury may manifest acutely—as severe left upper quadrant pain, hypotension, and signs of intraperitoneal hemorrhage—or insidiously, progressing to delayed complications such as splenic abscesses or infarctions.^20-22^ Recognizing this wide spectrum is paramount; early and ongoing postoperative monitoring using imaging modalities (CT and ultrasound) is often essential to guide timely management.

 Management strategies vary according to the grade of injury and the patient’s hemodynamic stability. For low-grade injuries, a conservative approach involving close observation, supportive care, and serial imaging has proven effective.^25-27^ In contrast, interventional radiology—specifically splenic artery embolization—offers an organ-preserving alternative for patients with localized vascular compromise.^28,29,31^ Ultimately, surgical intervention (whether via splenorrhaphy or splenectomy) remains the definitive management for severe injuries, though the loss of splenic function must be balanced against the immediate need to control hemorrhage.^24,30^

 Prevention is undoubtedly the most desirable strategy. Comprehensive preoperative planning—including detailed vascular imaging and risk stratification—coupled with refined intraoperative techniques (careful dissection, optimal retraction, and the use of adjuncts like indocyanine green fluorescence imaging) significantly reduces the likelihood of splenic injury.^14^ Equally critical are surgeon training, simulation exercises, and multidisciplinary collaboration, all of which promote adherence to standardized protocols and ultimately improve patient outcomes.^27^

 In summary, integrating these evidence-based strategies into clinical practice not only aids in the prompt recognition and effective management of splenic injury but also sets the stage for its prevention. Despite the information provided in this review, there are gaps across different aspects of splenic injury during sleeve gastrectomy, from potential risk factors to management protocols, particularly regarding standardized splenic injury grading specific to bariatric procedures. Therefore, future prospective studies and technological innovations—ranging from advanced energy devices to personalized surgical planning using three-dimensional reconstructions—are essential to further refine these approaches and enhance surgical safety in bariatric practice.^1,12,15,17,25^

 This review, consolidating insights from recent studies, provides clinicians with a detailed framework for recognizing, managing, and ultimately preventing splenic injuries during LSG. By adopting these comprehensive strategies, surgical teams can reduce morbidity and improve overall patient outcomes in bariatric surgery.

## References

[1] Ng M, Fleming T, Robinson M, Thomson B, Graetz N, Margono C (2014). Global, regional, and national prevalence of overweight and obesity in children and adults during 1980-2013: a systematic analysis for the Global Burden of Disease Study 2013. Lancet.

[2] Haslam DW, James WP (2005). Obesity. Lancet.

[3] Schauer PR, Kashyap SR, Wolski K, Brethauer SA, Kirwan JP, Pothier CE (2012). Bariatric surgery versus intensive medical therapy in obese patients with diabetes. N Engl J Med.

[4] Gloy VL, Briel M, Bhatt DL, Kashyap SR, Schauer PR, Mingrone G (2013). Bariatric surgery versus non-surgical treatment for obesity: a systematic review and meta-analysis of randomised controlled trials. BMJ.

[5] Sieber P, Gass M, Kern B, Peters T, Slawik M, Peterli R (2014). Five-year results of laparoscopic sleeve gastrectomy. Surg Obes Relat Dis.

[6] Stamou KM, Menenakos E, Gomatos IP, Panousopoulos SG, Smparounis S, Leandros E (2011). Clinical implications of sleeve gastrectomy as a source of spleen infarction or ischemia. Obes Surg.

[7] Belluzzi A, Sample JW, Marrero K, Tomey D, Puvvadi S, Sharma I (2024). Rare complications following laparoscopic sleeve gastrectomy. J Clin Med.

[8] Yesiltas MA, Gokkurt Y, Ketenciler S, Yucel C, Yilmaz M, Ozgol I (2024). Iatrogenic vascular injuries in elective abdominal and pelvic surgery patients: retrospective, single center, 30-day results. Sisli Etfal Hastan Tip Bul.

[9] Gulinac M, Miteva DG, Peshevska-Sekulovska M, Novakov IP, Antovic S, Peruhova M (2023). Long-term effectiveness, outcomes and complications of bariatric surgery. World J Clin Cases.

[10] Romeo L, Bagolini F, Ferro S, Chiozza M, Marino S, Resta G (2021). Laparoscopic surgery for splenic injuries in the era of non-operative management: current status and future perspectives. Surg Today.

[11] Delgado LM, de Souza VF, Pompeu BF, de Moraes Ogawa T, Oliveira HP, da Costa Sacksida Valladão V (2025). ​​Long-term outcomes in sleeve gastrectomy versus Roux-en-Y gastric bypass: a systematic review and meta-analysis of randomized trials. Obes Surg.

[12] Madhok B, Nanayakkara K, Mahawar K (2022). Safety considerations in laparoscopic surgery: a narrative review. World J Gastrointest Endosc.

[13] Petroianu A, Petroianu S (1994). Anatomy of splenogastric vessels in patients with schistosomal portal hypertension. Clin Anat.

[14] Frezza EE, Reddy S, Gee LL, Wachtel MS (2009). Complications after sleeve gastrectomy for morbid obesity. Obes Surg.

[15] Gürleyik E, Gürleyik G, Bingöl K, Akkaya L, Unalmişer S (2000). Perfusion and functional anatomy of the splenic remnant supplied by short gastric vessels. Am J Surg.

[16] Wu MP, Ou CS, Chen SL, Yen EY, Rowbotham R (2000). Complications and recommended practices for electrosurgery in laparoscopy. Am J Surg.

[17] Zhang X, Wei Z, Fu H, Hu Z, Wang W, Yan R (2024). Predictors of iatrogenic splenic injury in radical gastrectomy for gastric cancer. Front Oncol.

[18] Lalor PF, Tucker ON, Szomstein S, Rosenthal RJ (2008). Complications after laparoscopic sleeve gastrectomy. Surg Obes Relat Dis.

[19] Feola A, Niola M, Conti A, Delbon P, Graziano V, Paternoster M (2016). Iatrogenic splenic injury: review of the literature and medico-legal issues. Open Med (Wars).

[20] Sakran N, Zakeri R, Madhok B, Graham Y, Parmar C, Mahawar K (2021). Splenic abscess following sleeve gastrectomy: a systematic review of clinical presentation and management methods. Obes Surg.

[21] Mertens M, Verheyen L, Ceulemans J (2025). Spontaneous splenic rupture two days after laparoscopic sleeve gastrectomy. Acta Chir Belg.

[22] Shin CI, Kim SH (2020). Normal and abnormal postoperative imaging findings after gastric oncologic and bariatric surgery. Korean J Radiol.

[23] Blachar A, Federle MP, Pealer KM, Abu Abeid S, Graif M (2004). Radiographic manifestations of normal postoperative anatomy and gastrointestinal complications of bariatric surgery, with emphasis on CT imaging findings. Semin Ultrasound CT MR.

[24] Sandal M, Abou Hussein B, Buti F, Al Marzouqi O, Khammas A (2020). Spontaneous splenic rupture two weeks after sleeve gastrectomy. Obes Surg.

[25] Soriano-Giménez V, Ruiz de Angulo-Martín D, Munítiz-Ruiz V, de Los Ángeles Ortiz-Escandell M, Martínez-de Haro LF, Parrilla-Paricio P (2017). [Splenic late infarction after laparoscopic gastrectomy: a case report]. Cir Cir.

[26] Ertuğrul I, Yaylak F, Şenkul M, Atlı E, Tardu A (2018). Splenic infarction after laparoscopic sleeve gastrectomy. Laparosc Endosc Surg Sci.

[27] Mousavimaleki A, Amr B, Taherzadeh M, Rokhgireh S, Setaredan SA, Kermansaravi M (2022). Post-bariatric splenic complications; diagnosis and treatment A systematic review. Obes Surg.

[28] Vu T, Pilgrim C, Clements W, Ban EJ (2021). Delayed splenic abscess post splenic arterial embolization for trauma in a patient with previous sleeve gastrectomy. Clin Med Rev Case Rep.

[29] Roh S (2024). Splenic artery embolization for trauma: a narrative review. J Trauma Inj.

[30] Peters TG, Steinmetz SR, Cowan GS Jr (1990). Splenic injury and repair during bariatric surgical procedures. South Med J.

[31] Cheng J, Sun LY, Liu J, Zhang CW (2022). Indocyanine green fluorescence imaging for spleen preservation in laparoscopic splenic artery aneurysm resection: a case report. World J Gastrointest Surg.

[32] Rubin LG, Schaffner W (2014). Clinical practice Care of the asplenic patient. N Engl J Med.

